# Supplementary material for: Assessment of Maternal Exposure to Mycotoxins During Pregnancy Through Biomarkers in Fetal and Neonatal Tissues
Source: Toxins (Basel). 2025 Oct 21;17(10):518. doi: 10.3390/toxins17100518 (PMC12567740; doi:10.3390/toxins17100518)
Supplement: Supplementary file 1 [file toxins-17-00518-s001.zip › toxins-3874244-supplementary.pdf]

toxins

MDPI

Supplementary Materials

Table S1. Mass spectrometry parameters for determination of residual mycotoxins in liver tissue and serum samples.

Mycotoxin	Retention time (min)	Mass (g/mol)	Molecular ion	Transitions (*m/z*)	Cone voltage (V)	Collision energy (V)
AFM$_1$	4.25	328.3	[M+H]+	329.0 > 273.1 [a]	52	24
				329.0 > 229.0 [b]		
AFB$_1$	1.25	312.3	[M+H]+	312.7 > 284.9 [a]	94	36
				312.7 > 241.1 [b]		22
[$^{13}C_{17}$]-AFB$_1$	1.25	329.1	[M+H]+	330.3 > 301.5	94	20
AFB$_2$	1.15	314.3	[M+H]+	314.7 > 259.0 [a]	2	28
				314.7 > 287.0 [b]		26
AFG$_1$	1.18	328.3	[M+H]+	328.9 > 243.0 [a]	2	26
				328.9 > 199.5 [b]		38
AFG$_2$	1.16	330.3	[M+H]+	330.9 > 245.0 [a]	56	28
				330.9 > 188.9 [b]		40
OTA	5.79	403.1	[M+H]+	404.0 > 238.9 [a]	35	22
				404.0 > 357.9 [b]		12
[$^{13}C_{20}$]-OTA	5.79	423.7	[M+H]+	424.2 > 250.0	30	20
FB$_1$	5.40	721.8	[M+H]+	722.5 > 334.0 [a]	30	20
				722.5 > 352.1 [b]		40
[$^{13}C_{34}$]-FB$_1$	5.40	755.6	[M+H]+	756.6 > 374.4	50	30
FB$_2$	5.76	705.8	[M+H]+	706.5 > 336.2 [a]	50	35
				706.5 > 318.3 [b]		40
ZEN	6.03	318.1	[M-H]−	317.1 > 175.1 [a]	50	23
				317.1 > 130.9 [b]		33
[$^{13}C_{18}$]-ZEN	6.03	336.2	[M-H]−	335.1 > 185.1	50	31
α-ZEL	5.81	320.2	[M-H]−	319.1 > 275.2 [a]	30	20
				319.1 > 160.2 [b]		30
β-ZEL	5.83	320.2	[M-H]−	319.1 > 275.2 [a]	30	20
				319.1 > 160.2 [b]		30
DON	1.98	296.3	[M+H]+	297.3 > 249.1 [a]	15	10
				297.3 > 231.1 [b]		14
[$^{13}C_{15}$]-DON	1.98	311.2	[M+H]+	312.1 > 98.7	30	35
T-2 toxin	5.97	466.2	$[M+NH_4]^+$	484.2 > 305.2 [a]	20	10
				484.2 > 185.0 [b]	20	22
[$^{13}C_{24}$]–T-2 toxin	5.97	490.3	$[M+NH_4]^+$	484.2 > 305.2 [a]	20	10
HT-2 toxin	5.49	424.2	$[M+NH_4]^+$	442.2 > 263.3 [a]	20	10
				442.2 > 215.4 [b]		22

AFM$_1$: aflatoxin M$_1$; AFB$_1$: aflatoxin B$_1$; AFB$_2$: aflatoxin B$_2$; AFG$_1$: aflatoxin G$_1$; AFG$_2$: aflatoxin G$_2$; OTA: ochratoxin A; FB$_1$: fumonisin B$_1$; FB$_2$: fumonisin B$_2$; ZEN: zearalenone; α-ZEL: α-zearalenol; β-ZEL: β-zearalenol; DON: deoxynivalenol.

[a] transition used for quantification.

[b] transition used for quantification.

Table S2. Analytical parameters of the method used for determination of residual mycotoxins in liver tissue and serum samples from neonates and stillborn fetuses autopsied in Ribeirão Preto, Brazil.

Mycotoxin	Liver tissue samples			Serum samples		
	Calibration range (ng/g)	LOD (ng/g)	LOQ (ng/g)	Calibration range (ng/mL)	LOD (ng/mL)	LOQ (ng/mL)
AFM_1	0.10-100	0.10	0.30	0.06-4.00	0.06	0.20
AFB_1	0.10-100	0.10	0.30	0.06-4.00	0.06	0.20
AFB_2	0.10-100	0.10	0.30	0.06-4.00	0.06	0.20
AFG_1	0.10-100	0.20	0.60	0.06-4.00	0.06	0.20
AFG_2	0.10-100	0.20	0.80	0.06-4.00	0.06	0.20
OTA	0.10-100	0.10	0.30	0.06-4.00	0.14	0.48
FB_1	0.10-100	0.30	1.00	0.06-4.00	0.19	0.63
FB_2	0.10-100	0.40	1.00	0.06-4.00	0.19	0.63
ZEN	1.00-100	1.00	3.00	0.10-5.00	0.22	0.67
α-ZEL	1.00-100	0.80	2.60	0.10-5.00	0.18	0.70
β-ZEL	1.00-100	0.60	2.00	0.10-5.00	0.18	0.72
DON	5.00-100	2.00	6.00	0.60-20.00	1.12	3.20
T-2 toxin	1.00-100	0.90	2.80	0.10-5.00	1.25	2.80
HT-2 toxin	1.00-100	1.00	2.90	0.10-5.00	2.15	5.85

AFM_1: aflatoxin M_1; AFB_1: aflatoxin B_1; AFB_2: aflatoxin B_2; AFG_1: aflatoxin G_1; AFG_2: aflatoxin G_2; OTA: ochratoxin A; FB_1: fumonisin B_1; FB_2: fumonisin B_2; ZEN: zearalenone; α-ZEL: α-zearalenol; β-ZEL: β-zearalenol; DON: deoxynivalenol.

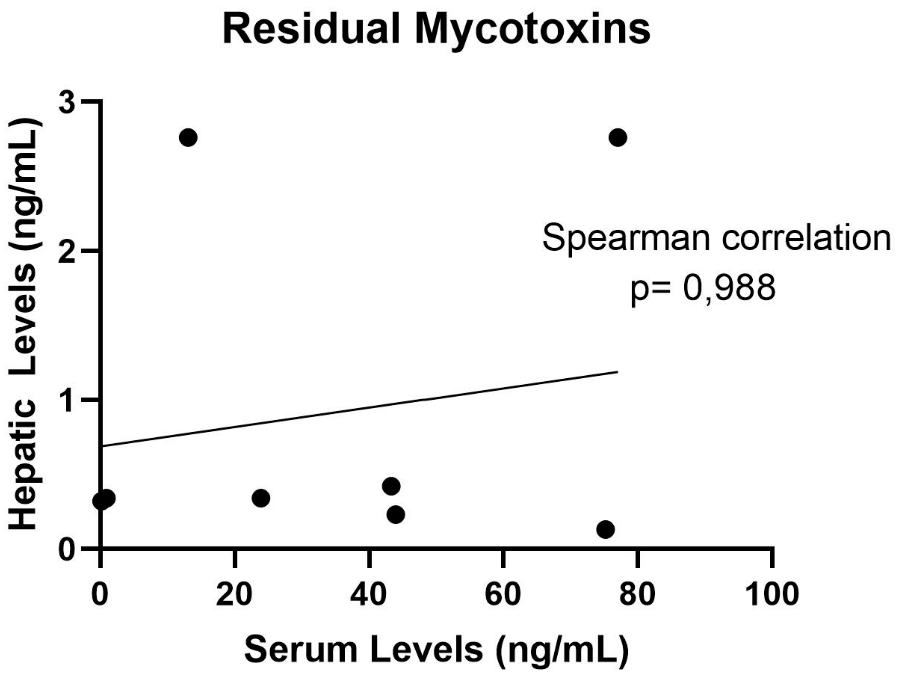

Figure S1. Correlation between the quantifiable levels of mycotoxins in the liver and serum samples from neonates and stillborn fetuses autopsied in Ribeirão Preto, Brazil ($n = 7$).
